# Definitive chemoradiation in vulvar squamous cell carcinoma: outcome and toxicity from an observational multicenter Italian study on vulvar cancer (OLDLADY 1.1)

**DOI:** 10.1007/s11547-023-01712-8

**Published:** 2023-09-12

**Authors:** Gabriella Macchia, Valentina Lancellotta, Martina Ferioli, Calogero Casà, Donato Pezzulla, Brigida Pappalardi, Concetta Laliscia, Edy Ippolito, Jacopo Di Muzio, Alessandra Huscher, Francesca Tortoreto, Mariangela Boccardi, Roberta Lazzari, Anna Myriam Perrone, Francesco Raspagliesi, Angiolo Gadducci, Giorgia Garganese, Simona Maria Fragomeni, Gabriella Ferrandina, Alessio Giuseppe Morganti, Maria Antonietta Gambacorta, Luca Tagliaferri

**Affiliations:** 1 Radiation Oncology Unit, Gemelli Molise Hospital, Università Cattolica del Sacro Cuore, 86100 Campobasso, Italy; 2 Dipartimento Di Diagnostica Per Immagini, Radioterapia Oncologica Ed Ematologia, UOC Radioterapia Oncologica, Fondazione Policlinico Universitario “A. Gemelli” IRCCS, 00168 Rome, Italy; 3 Radiation Oncology, IRCCS Azienda Ospedaliero-Universitaria Di Bologna, Bologna, Italy; 4 UOC Di Radioterapia Fatebenefratelli Isola Tiberina. Gemelli Isola, Rome, Italy; 5 Radiotherapy Unit, Fondazione IRCCS Istituto Nazionale Dei Tumori, Milan, Italy; 6 Division of Radiation Oncology, Department of New Technologies and Translational Research, University of Pisa, Pisa, Italy; 7 Radiation Oncology, Università Campus Bio-Medico, Rome, Italy; 8 Dipartimento Di Oncologia P.O. S. Anna - SS Radioterapia, A.O.U “Città Della Salute E Della Scienza”, Turin, Italy; 9 Fondazione Poliambulanza, U.O. Di Radioterapia Oncologica “Guido Berlucchi”, Brescia, Italy; 10 Division of Radiotherapy, IEO European Institute of Oncology IRCCS, Milan, Italy; 11 Division of Oncologic Gynecology, IRCCS Azienda Ospedaliero-Universitaria Di Bologna, Bologna, Italy; 12 Gynecologic Oncology, Fondazione IRCCS Istituto Nazionale Dei Tumori, Milan, Italy; 13 Division of Gynecology and Obstetrics, Department of Clinical and Experimental Medicine, University of Pisa, Pisa, Italy; 14 Dipartimento Scienze Della Salute Della Donna, del bambino e Di Sanità Pubblica, UOC Ginecologia Oncologica,, Fondazione Policlinico Universitario “A. Gemelli” IRCCS, 00168 Rome, Italy; 15 Dipartimento Scienze Della Vita E Sanità Pubblica, Sezione Di Ginecologia Ed Ostetricia, Università Cattolica del Sacro Cuore, Rome, Italy; 16 Department of Medical and Surgical Sciences (DIMEC), University of Bologna, Bologna, Italy; 17 Università Cattolica del Sacro Cuore Sede Di Roma, 00168 Rome, Italy

**Keywords:** Advanced vulvar cancer, Chemoradiation, Outcomes, Toxicity

## Abstract

**Background:**

Vulvar carcinoma is a rather uncommon gynecological malignancy affecting elderly women and the treatment of loco-regional advanced carcinoma of the vulva (LAVC) is a challenge for both gynecologic and radiation oncologists. Definitive chemoradiation (CRT) is the treatment of choice, but with disappointing results. In this multicenter study (OLDLADY-1.1), several institutions have combined their retrospective data on LAVC patients to produce a real-world dataset aimed at collecting data on efficacy and safety of CRT.

**Methods:**

The primary study end-point was 2-year-local control (LC), secondary end-points were 2-year-metastasis free-survival (MFS), 2-year-overall survival (OS) and the rate and severity of acute and late toxicities. Participating centers were required to fill data sets including age, stage, histology, grading as well as technical/dosimetric details of CRT. Data about response, local and regional recurrence, acute and late toxicities, follow-up and outcome measures were also collected. The toxicity was a posteriori documented through the Common Terminology Criteria for Adverse Events version 5 scale.

**Results:**

Retrospective analysis was performed on 65 patients with primary or recurrent LAVC treated at five different radiation oncology institutions covering 11-year time interval (February 2010–November 2021). Median age at diagnosis was 72 years (range 32–89). With a median follow-up of 19 months (range 1–114 months), 2-year actuarial LC, MFS and OS rate were 43.2%, 84.9% and 59.7%, respectively. In 29 patients (44%), CRT was temporarily stopped (median 5 days, range 1–53 days) due to toxicity. The treatment interruption was statistically significant at univariate analysis of factors predicting LC (*p*: 0.05) and OS rate (*p*: 0.011), and it was confirmed at the multivariate analysis for LC rate (*p*: 0.032). In terms of toxicity profile, no G4 event was recorded. Most adverse events were reported as grade 1 or 2. Only 14 acute G3 toxicities, all cutaneous, and 7 late G3 events (3 genitourinary, 3 cutaneous, and 1 vaginal stenosis) were recorded.

**Conclusion:**

In the context of CRT for LAVC, the present study reports encouraging results even if there is clearly room for further improvements, in terms of both treatment outcomes, toxicity and treatment interruption management.

## Introduction

Vulvar cancer is a rare disease affecting elderly women and representing about 5% of gynaecological cancers [1, 2]. The treatment of loco-regional advanced carcinoma of the vulva (LAVC) is a challenge for both gynaecologic and radiation oncologists.

Among the global new cases of vulvar cancers, approximately one third is represented by LAVC defined as a cancers which involve surrounding perineal organs (such as the urethra/bladder, upper vagina or rectum/anus), with unresectable inguinal nodes or in case of pelvic nodal disease [1, 2].

As per ESGO guidelines, definitive chemoradiadiation (CRT) is strongly recommended, while pelvic exenteratio/radical surgery, as well neoadjuvant chemoradiation followed by surgery may be considered [3]. Surgical procedures are associated with high operative mortality and postoperative physical and psychological morbidity [4, 5] with reported 4.3% mortality rate and 46% disease-free survival (DFS) [5]. On the other hand, definitive CRT compared with radical surgical approach allows organ preservation with good clinical outcomes and no significant differences in overall survival (OS) and DFS [6–11].

External beam radiation (RT), through image-guided and intensity-modulated treatments, has benefited from technical advancements in recent decades [12, 13]. The use of imaging techniques like computed tomography (CT), positron emission tomography (PET)-CT, and magnetic resonance imaging (MR) for treatment planning allows an accurate delineation of the tumor and organs at risk (OARs) with consequent better target dose coverage minimizing doses to healthy tissue [12, 13]. In the definitive scenario, dose escalation using modern RT techniques may improve tumor response and treatment tolerance [14, 15].

Due to the rarity of the disease, it is challenging to gather enough data to draw conclusions about any potential benefits of technique improvement. To overcome these limitations and to assess the outcomes in a larger case series of LAVC, we involved some Italian institutions that mostly deal with vulvar cancer.

The OLDLADY (ObservationaL Italian stuDy on vuLvar cAncer radical raDiotherapY) trials were approved and carried out by the Italian Association of Radiation Oncology's Gynecological group in collaboration with the Multicenter Italian Trials in Ovarian Cancer group and the Mario Negri Gynecologic Oncology Group [16].

Aim of the present paper was to assess the efficacy and safety of modern definitive CRT in LAVC.

## Material and methods

### Study design and end-points

The OLDLADY-1.1 trial pooled data on LAVC patients from five major Italian institutions. The primary study end-point was 2-year local control (LC), secondary end-points were 2-year-metastasis free-survival (MFS), 2-year OS and the rate and severity of acute and late toxicities.

### Procedures

For standardized data collection, the Principal Investigators (GM, LT) produced a dataset. The institutional, national research committee, and ethical criteria outlined in the Helsinki declaration were all followed during all operations. At the time of data collection, our institution did not require formal ethical approval for retrospective research. Data sets pertaining to age, stage, histology, grading, treatment break, and radiation technical/dosimetric features required to be completed by participants. LAVC were considered for enrollment if they had just been discovered or had returned after primary surgery. Clinical response, local and regional recurrences, acute and late toxicities, follow-up and outcome indicators, were all reported. Patients must have primary vulvar cancer that has been histologically confirmed, or recurrence after primary surgery, and they must have given their informed consent for treatment and the use of their clinical data for research. The scale for the Common Terminology Criteria for Adverse Events (CTCAE) version 5 was used a posteriori to evaluate the reported toxicity [17].

### Analysis of data and statistical methods

The study's radiation oncologists' medical databases were searched for relevant information. Information was also centrally gathered at the Radiation Oncology Departments of Gemelli Molise and Policlinico Gemelli IRCCS and entered an electronic database. The data processing was conducted by D.P., G.M., and V.L. On the patient, tumor, and therapy data, descriptive statistics were run.

The actuarial LC was defined as the time interval between the date of CRT start and the date of “in site” radiotherapy field relapse/progression/persistence of disease or the date of the last follow-up. The actuarial MFS was defined as the time interval between the date of CRT start and the date of out of field progression or the date of the last follow-up. The actuarial OS was defined as the time interval between the date of CRT start and the date of death or the date of the last follow-up. Actuarial outcomes were analyzed using Kaplan–Meier procedures; differences between subgroups were assessed using log-rank tests as well as univariate and multivariate Cox regression analysis. Treatment break (one or more days of treatment suspension versus none), complete remission to CRT (achieved vs. not achieved), age (< 65 vs. ≥ 65), stage (II vs. III–IV), lesion size (< 4 cm vs. ≥ 4 cm), grade (1 vs. 2 vs. 3), and primary lesion *versus* recurrence after primary surgery were the variables taken into account for the Cox regression analysis. Only variable with a *p* < 0.2 at univariate analysis were selected for the Multivariate analysis. The statistical analysis was done using SPSS (IBM Corp., 2011 release software. Version 20.0 of IBM SPSS Statistics for Windows. IBM Corp, Armonk, New York).

## Results

### Patient, tumor, and treatment characteristics

Sixty-five patients with primary or recurrent LAVC who were treated at five different radiation oncology facilities during an 11-year period (February 2010–November 2021) were analyzed. Age at diagnosis ranged from 32 to 89, with 72 being the median. Nine patients had relapses after surgery, while fifty-six patients were treated for initial tumors. The most frequent patient diagnoses were stage III (*N* = 31, 48%), grade 2 (*N* = 35, 54%), and squamous carcinoma (*N* = 65, 100%). 50 patients (77%) received CRT, as opposed to 15 patients (23%) who only received radiotherapy. Table 1 lists the features of the patient and the lesion.

**Table 1:** Patient and tumor characteristics of the study population

	*N*. (%)
All	65
Median age, years (range)	72 (32–89)
*Vulvar tumor* Primary Recurrence	57 (87.7%) 8 (12.3%)
AJCC prognostic stage group	IB: 3 (4.7%) II: 7 (10.8%) III A: 15 (23%) IIIB: 9 (13.8%) IVA: 15 (23%) IVB: 16 (24.7%)
Grading	G1: 10 (15.4%) G2: 35 (54%) G3: 5 (7.6%) Missing: 15 (23%)
Vulvar tumor size	< 4 cm: 17 (26.1%) ≥ 4 cm: 48 (79.9%)
Concomitant chemoradiation	Cisplatin: 30 (46%) Cisplatin plus 5-Fluorouracil: 15 (23%) Carboplatin: 3 (4.7%)
Temporary suspension of radiation treatment	29 (44.6%)

*AJCC* American Joint Committee on Cancer, *n* number

The median tumor total dose was 70 Gy (range 63–70.4 Gy). Negative inguinal/pelvic nodes received median total doses of 45/1.8 Gy fraction (range 45–50.4 Gy) while positive nodes received median total doses of 65/1.8 Gy fraction (range 50–70.4 Gy), respectively. Cisplatin alone (30 patients, 46%) or cisplatin plus 5-fluorouracil (15 patients, 23%) were the most used systemic agents for CRT. In detail, cisplatin (40 mg/m^2^, 2-h intravenous infusion once a week) or cisplatin (20 mg/m^2^, 2-h intravenous infusion, days 1–4) and 5-fluorouracil (1000 mg/m^2^, 24-h continuous intravenous infusion during the first and last weeks of radiotherapy) were administered.

### Treatment outcomes

At 3–4 months evaluation after CRT, a total of 43 patients (66.1%) showed disease complete remission. During follow-up 12 patients (18.4%) had vulvar or nodal recurrence, while 22 patients (33.8) presented persistence of disease. Nine patients (13.8%) developed distant metastases in lung (7.7%) and/or bone (6%). Median time to vulvar and nodal recurrence was 11 (range 6–46) and 15 (range 6–26) months, respectively.

**Fig. 1:**
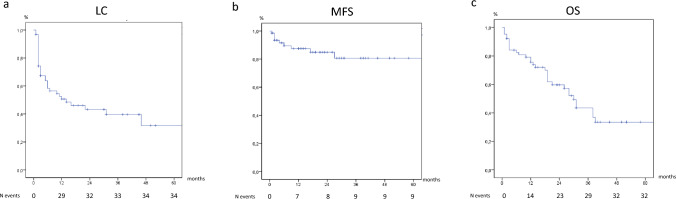
Kaplan–Meier curves: **1a** local control (LC); **1b** metastasis free-survival (MFS); **1c** overall survival (OS)

With a median follow-up of 19 months (range 1–114 months), 2-year actuarial LC rate was 43.2% (Fig. 1a). At the univariate analysis of factors predicting LC, only treatment interruption (HR 1.109, IC95%: 0.515–2.392, *p*: 0.05) and complete remission attainment (HR 432.0, IC95%: 5.0–33537.0, *p*: 0.006) could be used for the multivariate one, and only treatment interruption confirmed its statistical significance (HR 2.205, IC95%: 1.072–4.535, *p*: 0.032). As per MFS is concerned, 2-year actuarial MFS was 84.9% (Fig. 1b). The univariate analysis for variables predicting MFS rate showed no statistically significant results and no variables could be selected for the multivariate analysis. Lastly, 2-year OS was 59.7% (Fig. 1c). Univariate analysis of variables predicting OS rate showed that only the treatment interruption was statistically significant (HR 0.403, IC95%: 0.198–0.918, *p*: 0.011). No other variables could be selected for the multivariate analysis.

In terms of toxicity profile, no G4 event was recorded. Most of the reported events were reported as grade 1 or 2. Only 14 acute G3 toxicities were reported, all cutaneous ones, and 7 late G3 events (3 genitourinary ones, 3 cutaneous ones and 1 vaginal stenosis). More details are reported in Table 2.

**Table 2:** Acute and late toxicities

Acute toxicity	*N*	Late toxicity	*N*
All	69	All	54
GI		GI	
G1	2	G1	0
G2	5	G2	0
GU		GU	
G1	3	G1	1
G2	2	G2	6
		G3	3
Skin		Skin	
G1	4	G1	18
G2	29	G2	5
G3	14	G3	3
lymphoedema		lymphoedema	
G1	4	G1	6
G2	6	G2	4
		Vaginal stenosis	
		G1	3
		G2	4
		G3	1

G grade, *GI* gastrointestinal, *GU* genitourinary, *N* number

In 29 patients (44%), the radiation therapy was temporarily stopped (median 5 days, range 1–53 days) due to toxicity.

## Discussion

In a significant multi-institutional series of LAVC, definitive CRT is examined in the current paper. The major conclusions of this research highlight how the treatment of LAVC is difficult for radiation oncologists. Sixty-five cases of primary tumors and relapses were gathered over the course of 11 years by five different centers. Despite being a modest quantity, this mirrors a situation from the actual world [12].

Despite a median tumor and nodal total dose prescribed in accordance with international guidelines (NCCN 2023, ESGO 2022), the 2-year local control rate was 43.2% and the median time to relapse was below 15 months from treatment. Indeed, these figures are consistent with the efficacy phase II trial carried out by van Triest et al., who reported 42% 2-year LC and 58% 2-year regional control, nevertheless they conceal a potential inability to sterilize the irradiated zone, leading us to believe that this district is relatively undertreated [18].

Interestingly, roughly one out of four treated subjects failed to receive concomitant chemotherapy, underlining once again how toxicity, comorbidity and/or frailty of the patient may influence treatment planning. Among patients receiving concomitant chemotherapy about half received a single drug while about a quarter of the patients were treated with the doublet. There are few similar studies that can be compared to our analysis. The largest series is the one of Rao et al., who reported on 1352 patients with unresectable tumors who had radiation or CRT and were included in a comprehensive retrospective analysis based on the National Cancer Database [19]. The median dose of radiation was 59.40 Gy, which is less than what reported in the present study. Moreover, 62% of the CRT cohort received only one drug, whereas the remaining patients were given a multiagent platin-based regimen. Authors reported that CRT considerably outperformed radiotherapy in terms of 5-year OS, with stage II–IV disease showing the greatest benefit. Other small studies showed as a definitive combined treatment (radiotherapy *plus* chemotherapy) resulted in an improvement of DFS [20], disease-specific survival [20], and OS rates compared to patients treated with definitive radiation therapy alone [12, 19–22]. This benefit remained significant after adjusting for different factors such as age, race, performance status and FIGO stage [21]. Therefore, every effort should be made to potentiate radiotherapy with the help of sensitizing chemotherapy both in the multidisciplinary clinical decision-making phase and in the management of toxicities during and after treatment. However, in very elderly or frail patients, prospective and retrospective studies recommend caution for CRT because of treatment-related deaths [11, 23]. Therefore, it is not surprising that definitive CRT may not prolong OS in patients with multiple comorbidities. A multidisciplinary team is needed to allow a personalized management focusing on efficacy, feasibility, and cost/benefit considering the patient’s age, clinical condition, type of disease and personal needs [24–27].

In terms of adverse effects, CRT is frequently associated with acute cutaneous toxicity but with a low incidence of severe events. In our series, the treatment was well tolerated, especially when compared to previous studies where toxicities greater than grade 2 ranged from 25 to 50% in the definitive setting [12]. Indeed, skin toxicity may be a barrier to adequate radiation administration, thus it is of utmost importance to create preventive and supportive therapy regimens may be helpful in the management of this tough treatment.

Another remarkable aspect of this multi-center retrospective data is the interruption of CRT. It is well known the detrimental effect of treatment duration on LC in many neoplasms and above all in squamous cell tumors. For example, in the intact setting, prolonged treatment time for squamous cell carcinomas of the head and neck as well as cervical cancers have been found to lead to inferior outcomes as well [28, 29]. In our series, interruptions were critical with about half of the patients temporarily discontinuing RT/CRT (median 5 days, range 1–53 days), which might have contributed to the poor LC outcomes. Furthermore, only the treatment interruption was statistically significant at univariate analysis of factors predicting LC (*p*: 0.05) and OS rate (*p*: 0.011), and it was confirmed at the multivariate analysis for LC rate (*p*: 0.032). These findings might be explained by the high *D*_prolif_ (i.e., the dosage required to compensate for one day of treatment suspension) of vulvar cancer, which would need dose recovery for the break. Due to the retrospective design of the study, no data are available concerning dose recovery policy in the involved radiation Centers. Regarding the impact of chemoradiation complete remission on LC, the loss of the statistical significance at the multivariate analysis could be explained with the limited sample size.

In the present series, 2-year actuarial MFS and OS were 84.9% and 59.7%, respectively. When read in the context of often elderly, frail, and comorbid patients, these findings explain why definitive CRT should be considered the standard of care in the management of patients with locally advanced vulvar carcinoma who are not candidates for primary surgery due to unresectable disease or poor performance status [3, 11, 21, 23, 30]. In comparison with surgery, definitive CRT allows for organ preservation while still providing acceptable clinical results. Furthermore, surgery may have a harmful effect on physical/psychological aspects in LAVC patients, and surgical mortality is not negligible [4, 31, 32]. Perioperative complications such as wound dehiscence, infection, and seroma might delay the start of adjuvant treatment, impacting on LC and OS [30]. A retrospective study of 63 patients with stage III-IV carcinoma of the vulva showed no significant differences in OS and DFS according to treatment group (CRT vs. surgery; *p* = 0.83, *p* = 0.81, respectively) [21]. A systematic review showed no differences in terms of survival and treatment-related adverse effects in primary surgery group when compared to CRT for women with LAVC [22].

Due to the retrospective study design, which affected data collection, treatment, and follow-up, as well as the small number of occurrences, this study had substantial limitations, including the potential for hidden bias. Age, radiation dose, and stage most likely had no effect on LC and OS rates for the limit specified above. The absence of information regarding the status of the human papillomavirus (HPV) was another restriction. In a recent publication, Horne and colleagues hypothesized that tumors that are p16-positive exhibit superior clinical and pathologic response rates as well as clinical outcomes. The authors found that women with p16 + tumors had a 2-year LC of 75.5% and a complete clinical response rate of 63.6% compared to 35.0% for p16 tumors (*p* = 0.014) and a 2-year LC of 75.5% for women with *p*16 + tumors versus 49.5% for *p*16 − (*p* = 0.008) [33].

Instead, one of the paper's strengths is how many Italian institutions working together to combat this disease have paved the road for future multi-center prospective research. Additionally, we have included our case series in the retrospective studies that emphasize the value of conclusive care in this situation.

Patients with vulvar cancer should be treated in experienced centers (i.e., large patient volume and updated treatment technique) due to the rarity of the disease and absence of randomized studies because it affects OS [31]. To provide individualized care and improve clinical results, the decision to proceed with definitive CRT rather than primary surgery should be made in a multidisciplinary environment on an individual basis [25, 34].

## Conclusion

The current study reveals positive findings, even if there is obviously space for improvement in terms of treatment outcomes, toxicity, and management of treatment cessation. These developments could come through prospective and possibly randomized studies, despite the disease rarity severely limiting their viability. Alternately, or additionally, the production of prediction models may be made possible by the development of massive data repositories. These models are especially useful for designing tailored therapy based on the characteristics of the patient and the tumor [34–37]. The most effective therapeutic approach at the moment is multimodality treatment based on multidisciplinary debate in the tumor board of LAVC patients to choose the best course of action for the specific subject.
